# Impaired Bone Physiology in Rheumatoid Arthritis: Can the Wnt Pathway be the Connecting Link Between the Synovium and the Bone?

**DOI:** 10.31138/mjr.140925.eri

**Published:** 2026-04-21

**Authors:** Michail I. Krikelis, Dimitra Moschou, Konstantina Zoupidou, Evangelia Mole, Konstantinos Makris, Simeon Tournis, Sousana Gazi, Vassilios Nikolaou, Clio Mavragani, Efstathios Chronopoulos

**Affiliations:** 1 Laboratory for the Research of Musculoskeletal System “Th. Garofalidis”, Medical School, National and Kapodistrian University of Athens, KAT General Hospital, Athens, Greece;; 2 Rheumatologist, Larnaka, Cyprus;; 3 Attikon University Hospital, Rheumatology and Clinical Immunology Unit, 4th University Clinic of Internal Medicine, Athens, Greece;; 4 Rheumatology Department, KAT General Hospital, Athens, Greece;; 5 Department of Biochemistry, KAT General Hospital, Athens, Greece;; 6 2nd University Clinic of Orthopaedic Surgery, Konstantopoulio General Hospital, Athens, Greece;; 7 Laboratory of Experimental Physiology, School of Medicine, National and Kapodistrian University of Athens, Athens, Greece;; 8 Laboratory for the Research of Musculoskeletal System “Th. Garofalidis”, Medical School, National and Kapodistrian University of Athens, KAT General Hospital, Greece, Athens, Greece

**Keywords:** rheumatoid arthritis, bone loss, Wnt pathway

## Abstract

**Introduction and Methods:**

Rheumatoid arthritis (RA) is a chronic inflammatory disease characterised by bone loss. A Medline/PubMed search was conducted in September 2025 using the keywords “rheumatoid arthritis” AND “bone loss.” The search was restricted to recently published review or systematic review articles (2020–2025). In total, 24 articles were thoroughly reviewed.

**Results:**

Inflammation-induced accelerated bone resorption both at the systemic level and in juxta-articular bone is typical to rheumatoid arthritis. Bone formation and bone biomechanics are also impaired due to inflammation, prolonged or repeated corticosteroid treatment, and muscle wasting.

**Conclusions:**

Aberrant bone loss and impaired bone formation are hallmarks of rheumatoid arthritis. The underlying pathophysiological mechanisms differ between early and refractory disease, suggesting a potential central role of the highly conserved Wnt pathway in the bone impairment observed in established RA.

## INTRODUCTION AND METHODS

Rheumatoid arthritis (RA) is a chronic autoimmune condition with an estimated global prevalence of 208.8 cases per 100,000 population (0.2%).^1^ The aberrant activation of the immune system causes prolonged inflammation in articular, peri-articular, and extra-articular tissues, leading to irreversible damage and disability.

Bone loss is a common finding in patients with RA and typically occurs on three levels: juxta-articular bone loss and bony erosions, peri-articular bone demineralisation, and systemic bone loss, resulting in conditions such as osteopenia or osteoporosis.^2^ It is now understood that in autoimmune diseases like RA, the immune system interferes with bone tissue metabolism through various pathophysiological pathways, accelerating bone resorption and inhibiting bone formation. This emerging understanding has given rise to a novel field of study called “osteoimmunology” or “immunoporosis” which investigates the immune-bone interactions to elucidate the mechanisms that contribute to bone loss in autoimmune diseases.^3^

The present review contributes to this growing body of knowledge by using RA as a model autoimmune disease characterised by bone loss. It explores the most critical pathophysiological mechanisms that lead to decreased bone mass and quality in RA. However, it takes a novel approach by viewing bone tissue not only as a target of immune dysfunction but also as a dynamic and functional tissue capable of influencing immune responses. Thus, it seeks to delineate the principal pathways through which this interplay occurs, providing fresh insights into the relationship between bone tissue and the immune system. Special attention is paid to the pathophysiological role of the Wnt pathway in light of the recent development of promising monoclonal antibody therapies already used in the treatment of postmenopausal osteoporosis.

For the purposes of this article, a search was conducted in the Medline/PubMed database in September 2025 using the following keywords: “rheumatoid arthritis” AND “bone loss”. The search was restricted to recently published articles (2020–2025) that included a review or systematic review on the topic. A total of 24 articles met these criteria. These articles were thoroughly reviewed, and the conclusions are presented below.

### Increased bone loss in rheumatoid arthritis

Bone homeostasis is maintained through a tightly regulated coupling of osteoclast and osteoblast functions, primarily mediated by the RANK/RANKL/OPG pathway. RANK (Receptor Activator of Nuclear Factor-kappa B) is a receptor on the surface of osteoclast precursors and mature osteoclasts. It belongs to the tumour necrosis factor (TNF) receptor superfamily, and its activation facilitates osteoclast differentiation, survival, and activity. RANKL, a ligand produced by osteoblasts, osteocytes, and stromal cells, binds to RANK on osteoclast precursors, promoting differentiation into mature, multinucleated osteoclasts capable of bone resorption. OPG (osteoprotegerin), a decoy receptor secreted by osteoblasts and other cells, binds to RANKL, preventing it from interacting with RANK. This suppresses osteoclastogenesis and limits bone resorption.^4^

In RA, a break in immune tolerance can occur up to 10 years before the onset of disease symptoms such as synovitis and malaise. This pre-clinical phase is often characterised by the overproduction of autoantibodies in up to 70% of patients with the seropositive form of the disease. The hallmark autoantibodies of RA are anti-citrullinated protein antibodies (ACPA), which are strongly associated with the presence of the disease.^5^ ACPA bind directly to citrullinated proteins on the membranes of osteoclast precursors, promoting their maturation.^6^ Additionally, ACPA interact with tissue macrophages, stimulating the production of TNF-alpha, which in turn enhances RANKL production by T- and B-lymphocytes as well as synovial fibroblasts.^7^ These mechanisms contribute to general bone loss, which may precede the onset of articular symptoms in seropositive RA patients.^8^

In the clinical phase, RA is marked by an inflammatory articular microenvironment that sustains a self-perpetuating network of RANKL production, particularly by synovial fibroblasts. Pro-inflammatory cytokines, including TNF-α, IL-1, IL-6, and IL-17, play key roles in this process. TNF-α independently promotes osteoclast maturation by upregulating the osteoclast-associated receptor (OSCAR).^9^ It also induces IL-1 production, which prolongs osteoclast survival by inhibiting apoptosis.^10^ IL-6 increases osteoclastogenesis and stimulates IL-17 production by TH17 T-lymphocytes, which further enhances RANKL secretion and osteoclast differentiation.^11,12^

This inflammatory cascade within the joints leads to the development of juxta-articular erosions due to increased osteoclast-mediated bone resorption. Additionally, the sustained overproduction of RANKL results in systemic bone resorption, particularly in areas rich in trabecular bone.^13^

The para-articular bone tissue in RA patients undergoes changes that favour increased bone resorption. Para-articular bone demineralisation is a common finding, with histological evidence showing increased resorption surfaces and numerous mature osteoclasts.^14^ Although distant from the synovial space, the pro-inflammatory environment within the bone marrow promotes osteoclast activity in para-articular bone. Furthermore, immobilisation caused by pain and articular inflammation contributes to bone loss by reducing mechanical stimuli necessary for maintaining bone integrity.

Glucocorticoids, often used to manage acute inflammation in RA, have significant effects on bone resorption. In the short term, glucocorticoids suppress osteoblast activity and promote apoptosis of osteoblasts and osteocytes. They also increase RANKL expression by osteoblasts and stromal cells while reducing OPG production, thereby accelerating osteoclastogenesis. Additionally, glucocorticoids extend the lifespan of osteoclasts, further enhancing bone resorption. Prolonged glucocorticoid therapy exacerbates bone fragility by reducing overall bone remodelling and promoting osteocyte apoptosis. These dose-dependent effects underscore the critical need for careful management of glucocorticoid therapy in RA patients to minimise bone loss and associated complications.^15^

To summarise, RA-associated bone loss arises from a complex interplay of autoantibodies, inflammatory cytokines, synovial fibroblasts, and treatment side effects. The imbalance between RANKL and OPG drives pathological osteoclastogenesis, resulting in both local erosions and systemic osteoporosis. Careful management of inflammation and glucocorticoid use is essential to protect bone integrity in RA patients.

### Impaired bone deposition in rheumatoid arthritis

The systemic inflammation observed in rheumatoid arthritis (RA) also impairs bone formation. Osteoblastogenesis is disrupted through altered sclerostin and Dkk-1 concentrations, which enhance inhibition of the Wnt signalling pathway.^16^

Changes in bone biomechanics have been demonstrated in biopsies from RA patients undergoing joint replacement surgery, showing excessive osteocyte apoptosis, high sclerostin expression, and empty osteocyte lacunae.^17^ Experimental studies on human bone tissue further indicate that both immature osteoblasts and osteocytes produce sclerostin under inflammatory conditions mediated by TNF-α family cytokines.^18^

Similarly, mouse models of TNF-α–mediated arthritis suggest that fibroblast-like synoviocytes increase sclerostin production under the paracrine effects of excessive TNF-α.^19^ However, deletion of the SOST gene or administration of sclerostin-neutralising antibodies in mice does not prevent juxta-articular bone erosions, which are only reversed by targeted anti-TNF-α therapy.^19–21^

In humans, the role of the Wnt pathway and its inhibitors remains less clear. Data regarding sclerostin levels in RA patients are contradictory, with discrepancies likely explained by differences in study design, recruitment methods, disease activity and duration, as well as age and menopausal status. In the large French ESPOIR cohort of early RA, elevated serum Dkk-1 (another internal inhibitor of the Wnt pathway) was identified as a biomarker predicting structural progression over two years.^22^ A prospective study of 27 treatment-naïve patients with early RA (<5 years) also demonstrated increased baseline Dkk-1 levels, which declined following methotrexate therapy.^23^

Biological therapies appear to partially restore bone homeostasis by reducing systemic inflammation, suppressing bone resorption, and promoting osteoblastogenesis. In a prospective study of 31 women with early active RA (<5 years), plasma sclerostin significantly decreased after 15 months of anti-TNF-α therapy.^24^ Treatment with IL-6 inhibitors (tocilizumab) has also been shown to reduce serum sclerostin in early drug-naïve RA.^25^

However, bone pathophysiology seems more complex in patients with long-standing disease. In drug-experienced RA patients with a mean disease duration of 7 years, 12 weeks of etanercept therapy did not normalise serum sclerostin, which remained elevated at levels comparable to those in early disease.^26^ Likewise, two months of tocilizumab treatment did not reduce serum sclerostin in women with a 10-year disease history.^27^ These findings suggest that prolonged inflammation and corticosteroid exposure may cause permanent alterations in bone biology, with additional mechanisms beyond inflammation contributing to impaired bone responses in chronic RA.

In short, systemic inflammation in RA suppresses osteoblastogenesis via Wnt pathway inhibition, primarily through sclerostin and Dkk-1. While biological therapies can restore bone homeostasis in early disease by lowering these inhibitors, long-standing RA shows persistent dysregulation, suggesting irreversible alterations in bone biology beyond inflammation alone.

### The Wnt pathway in reduced bone synthesis and muscle wasting

Beyond bone and cartilage, sclerostin also regulates muscle development. In bones not subjected to physical loading, sclerostin is secreted by osteocytes to suppress bone formation (osteoblastogenesis and mineralization) while enhancing bone resorption (osteoclast differentiation and maturation). At the same time, sclerostin functions as a cytokine with paracrine (osteokine) effects on neighbouring muscle tissue, where it suppresses the Wnt signalling pathway necessary for myogenesis. Reciprocally, muscle cells produce their own sclerostin, acting paracrinally (myokine) on adjacent bone.

Physical activity reduces sclerostin production by osteocytes through increased mechanical loading, thereby promoting bone formation. Simultaneously, reduced sclerostin levels in the muscular environment enhance the activity of Wnt3 and Wnt4 ligands, favouring satellite cell activation and muscle fibre formation.^28^ Indeed, osteocyte cultures have been shown to induce myogenesis and contractile function,^29^ while myoblasts in vitro also secrete sclerostin.^30^

This bidirectional muscle–bone crosstalk creates an anabolic loop sustained by physical activity, which is disrupted in pathological conditions affecting both tissues. Sarcopenia (loss of lean muscle mass) and osteosarcopenia (the coexistence of osteoporosis and sarcopenia) are emerging areas of research in ageing populations. The deterioration of both muscle and bone, together with the vicious cycle of reduced physical activity and increased frailty observed in older individuals, may be mediated by the suppression of Wnt signalling through sclerostin in both tissues.^31,32^

### Towards understanding bone metabolism in rheumatoid arthritis

How is all the above relevant to what happens to bone in rheumatoid arthritis? Can we develop an integrated theory that explains systemic and juxta-articular bone loss, bony erosions, reduced bone deposition, and muscle degradation? Could the Wnt pathway be the connecting link mediating this bone–synovium–muscle crosstalk?

In rheumatoid arthritis (RA), the aberrant inflammatory environment drives pathological changes not only in the joints but also in systemic bone metabolism and muscle integrity. In the early stages of the disease, osteoclasts become hyperactivated both directly, through binding of anti-citrullinated protein antibodies (ACPAs), and indirectly, through excess RANKL production. This leads to systemic bone loss and juxta-articular bone degradation. At the same time, articular inflammation promotes the overproduction of sclerostin by synovial-like fibroblasts and the overexpression of Dkk-1 by osteocytes. These Wnt pathway inhibitors act in a paracrine manner to suppress osteoblast activity and to reduce bone deposition in the juxta-articular bone. They may also contribute to the muscle wasting frequently observed in RA patients, pointing toward shared signalling pathways that link muscle and bone metabolism.^33^ Importantly, both sclerostin and Dkk-1 are also secreted into the circulation, suggesting a potential endocrine role that could connect synovial inflammation, systemic osteoporosis, and skeletal muscle degradation.

These pathophysiological mechanisms—osteoclast hyperactivation, impaired osteoblast function, and Wnt pathway inhibition—are mirrored in the altered serum bone turnover markers observed in early RA, where elevated concentrations of sclerostin and Dkk-1 are consistently detected. Treatment with corticosteroids, csDMARDs, or targeted biologics does restore bone homeostasis in patients who respond adequately to therapy. However, up to one-third of patients remain refractory to treatment.^34^ In these individuals, persistent inflammation, repeated glucocorticoid exposure, and long periods of immobility due to pain and fatigue create a vicious cycle: inflammation drives osteoclast activation and suppressed bone formation, which contribute to osteoporosis; reduced mobility worsens sarcopenia; and progressive muscle wasting further fuels fragility.

Within this cycle, the Wnt pathway appears to play a central role. Sclerostin is secreted not only by bone tissue but also by synovium and muscle, exerting both paracrine and potentially endocrine effects. This may explain why elevated serum sclerostin often persists despite biological therapy in patients with advanced disease. An integrated model of RA bone pathology can therefore be envisioned as an inflammatory–Wnt pathway dysfunction axis, in which inflammatory cytokines drive sclerostin and Dkk-1 expression, leading to suppressed bone formation, systemic bone loss, and muscle wasting.

From a therapeutic perspective, this model highlights the importance of early intervention. Combining anti-inflammatory treatments with strategies that target sclerostin could prevent both bone and muscle loss, thereby breaking the self-sustaining cycle of inflammation, sarcopenia, and fragility that characterises refractory RA.
